# Frequent *RNF43* mutation contributes to moderate activation of Wnt signaling in colorectal signet-ring cell carcinoma

**DOI:** 10.1007/s13238-020-00691-0

**Published:** 2020-02-01

**Authors:** Yaqi Li, Jian Li, Renjie Wang, Long Zhang, Guoxiang Fu, Xueying Wang, Yebin Wang, Chuantao Fang, Dandan Zhang, Duo Du, Xiaoji Ma, Mengxue Pan, Qiang Guo, Xiaoya Xu, Xiang Hu, Yi Zhou, Shaobo Mo, Huijun Wang, Jianjun Gao, Shenglin Huang, Yun Liu, Sanjun Cai, Guoqiang Hua, Junjie Peng, Fa-Xing Yu

**Affiliations:** 1 Department of Colorectal Surgery, Fudan University Shanghai Cancer Center, Shanghai, 200032 China; 2 Department of Oncology, Shanghai Medical College, Fudan University, Shanghai, 200032 China; 3 Institute of Pediatrics, Children’s Hospital of Fudan University, Shanghai, 200032 China; 4 Key Laboratory of Medical Epigenetics and Metabolism, Institutes of Biomedical Sciences, Fudan University, Shanghai, 200032 China; 5 Cancer Institute, Fudan University Shanghai Cancer Center, Fudan University, Shanghai, 200032 China; 6 Institute of Radiation Medicine, Fudan University, Shanghai, 200032 China; 7 MOE Key Laboratory of Metabolism and Molecular Medicine, Department of Biochemistry and Molecular Biology, School of Basic Medical Sciences, Fudan University, Shanghai, 200032 China; 8 Fudan University Shanghai Cancer Center and Institutes of Biomedical Sciences, Shanghai Medical College, Fudan University, Shanghai, 200032 China; 9 Institute of Radiation Medicine and Fudan University Shanghai Cancer Center, Shanghai Medical school, Fudan University, Shanghai, 200032 China

**Dear Editor,**

Signet-ring cell carcinoma (SRCC) is a rare subtype of colorectal cancer (CRC) characterized histologically by the accumulation of mucins in the cytoplasm and displacement of nuclei to the cellular periphery, accounting for about 1% CRC (Fig. S1A) (Borger et al., 2007). Compare to common subtypes of CRC, such as adenocarcinoma (AC) and mucinous adenocarcinoma (MAC), SRCC is associated with aggressive behaviors and younger age at presentation (Kang et al., 2005; Sung et al., 2008; Nitsche et al., 2013; Hugen et al., 2014; Inamura et al., 2015). A retrospective analysis of CRC patient’s data at Fudan University Shanghai Cancer Center (FUSCC) also indicated a worse overall and disease-free survival of SRCC patients (Fig. S1B and S1C, Table S1). Due to low incidence and occasionally mixed presence of SRCC with AC or MAC, genome-wide characterization of SRCC at a large scale is challenging. Limited whole-exome sequencing (*n* = 5) and gene panel sequencing (*n* = 35) results indicate that, most driver mutations associated with CRC, such as *APC*, *KRAS*, and *PIK3CA*, are mutated at lower rates in SRCC (Nam et al., 2018; Korphaisarn et al., 2019). Mutations and signaling pathways responsible for the tumorigenesis of SRCC remains to be uncovered.

Following microscopic analysis of 4,000 CRC specimens, we identified 29 SRCCs with a high percentage (>70%) of signet-ring cells. To gain a better understanding of genomic alterations in SRCC, we performed WES on these SRCCs and paired normal tissues (Tables S2 and S3). WES data of AC and MAC from the Cancer Genome Atlas (TCGA) were analyzed for comparison (Tables S4 and S5) (TCGA, 2012).

We identified 9,752 non-silent somatic mutations in SRCC samples, with dominant C>T/G>A substitutions enriched at CpG islands (Fig. S2 and Tables S6–9). The overall mutation rate of SRCC is 9.65/Mb (medium mutation rate is 3/Mb), and 3 cases (10.3%, with *POLE* mutation or microsatellite instability-high) were considered as hypermutated (Fig. 1A and Table S3). In total, there were 34 recurrent alterations, with *TP53* (55.2%), *RNF43* (34.5%), *MUC16* (31.0%), *TTN* (31.0%), *PCDH17* (27.6%), *KMT2D* (24.1%), and *SMAD4* (20.7%) as most frequently mutated genes (Fig. 1B and Table S10). Mutations on critical cancer drivers were validated by Sanger sequencing (Fig. S3 and Table S11). By comparing mutated genes in different cancer signaling pathways, we noticed that genes in p53 (e.g., *TP53*) and TGF-β (e.g., *SMAD4*) pathways were mutated at similar frequencies cross CRC subtypes, however the mutation burden in WNT, MAPK, and PI3K pathways were dramatically lower in SRCC, suggesting subtype-specific molecular signatures (Figs. 1C and S4).

**Figure 1:**
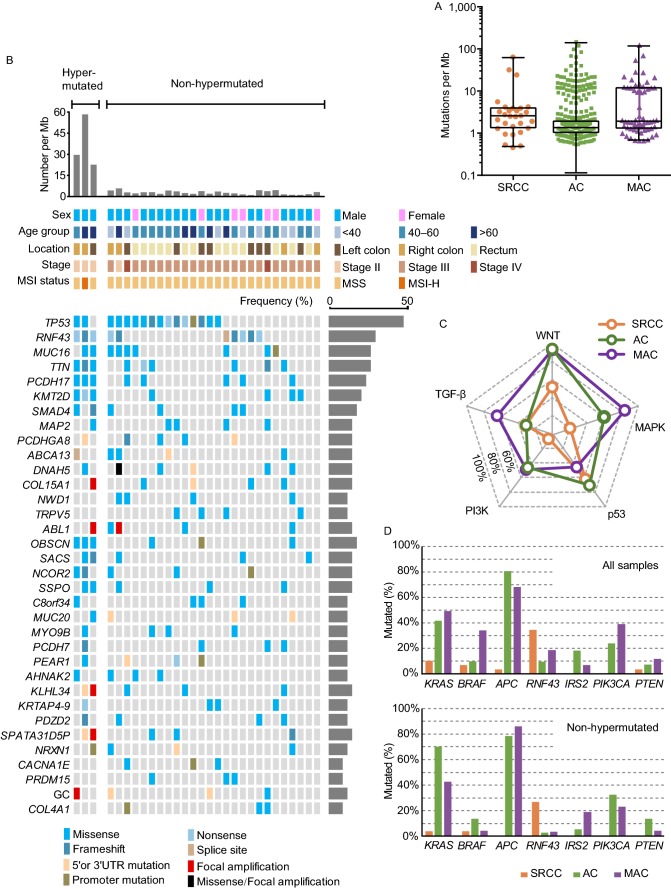

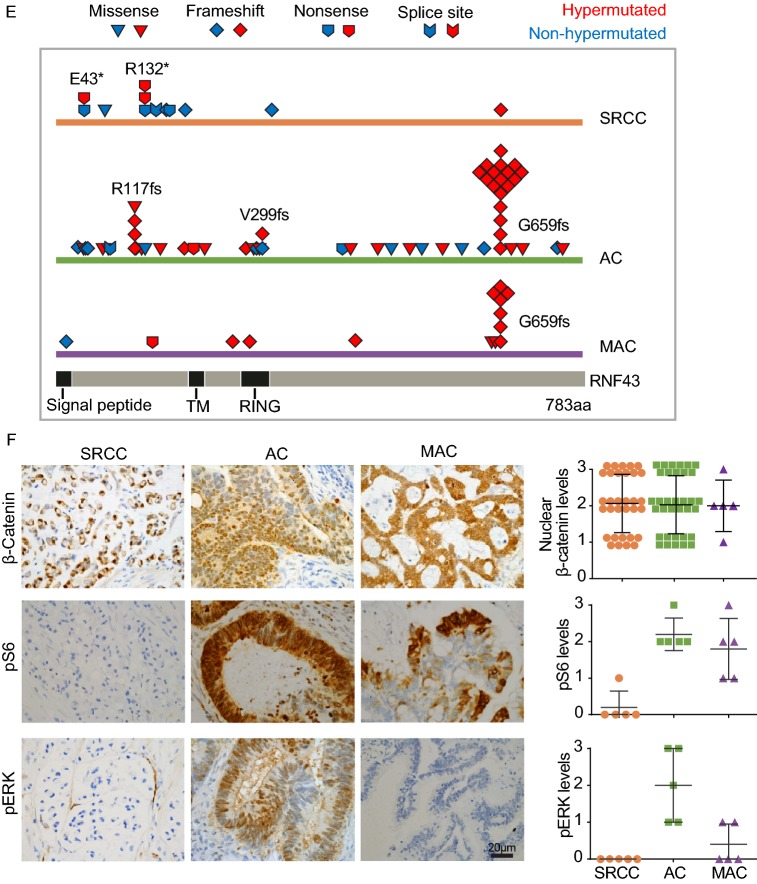
**WES and pathway analysis of colorectal SRCC**. (A) The tumor mutation burden (TMB) in colorectal SRCC (*n* = 29) from FUSCC and adenocarcinoma (AC, *n* = 458) and mucinous adenocarcinoma (MAC, *n* = 59) from the Cancer Genome Atlas (TCGA) cohort. (B) Top mutated genes and mutational landscape in 29 samples of colorectal SRCC. Different types of mutation were distinguished by colors. Frequency of each significant alteration was shown on the right of the heatmap. TMB, demographic, and clinical information for each patient were illustrated on the top of the heatmap. (C) Comparison of the alteration frequencies of top CRC-associated signaling pathways in SRCC, AC and MAC. (D) Comparison of mutation rates of cancer drivers in SRCC, AC, and MAC, all samples and non-hypermutated samples were analyzed. (E) Distribution and types of *RNF43* mutations in SRCC, AC and MAC. (F) The activating status of WNT (β-catenin), MAPK (pERK Thr202/Tyr204) and PI3K (pS6 Ser240/244) pathways were assessed by immunohistochemistry (IHC, left), and quantified (right). Scale bar, 20 μm

The WNT pathway is instrumental to intestinal homeostasis and the initiation of AC (Nusse and Clevers, 2017). A striking difference between SRCC and AC/MAC was the mutated genes in the WNT pathway (Figs. 1C,1D, S4 and S5). *APC* mutation was most prevalent in AC (~80%) and MAC (~70%) (TCGA, 2012), whereas it only occurred in one (3.4%) hypermutated SRCC, this is consistent with recent reports (Nam et al., 2018; Korphaisarn et al., 2019). Interestingly, SRCC was associated with frequent mutations in *RNF43*, with nonsense mutations (p.Glu43* and p.Arg132*) enriched at the N-terminus, regardless of mutation burden (Fig. 1D and 1E). In AC/MAC, most *RNF43* mutations occurred in hypermutated tumors, with a hotspot at C-terminal (p.Gly659 frameshift) (Fig. 1E and Table S12) (Giannakis et al., 2014; Yan et al., 2017). Both APC and RNF43 are key regulators of the WNT pathway, their inactivation leads to stabilization and nuclear translocation of β-catenin (Nusse and Clevers, 2017). Almost all SRCC, AC, and MAC samples showed discernable nuclear staining of β-catenin by immunohistochemistry (IHC) (Fig. 1F). It appeared that, different mechanisms are employed by CRC subtypes to activate β-catenin, in which SRCC prefers a complete inactivation of *RNF43* (N-terminal nonsense mutation), whereas AC and MAC prefer *APC* mutation. In addition, *DKK4* amplification and mutations on *FZD10*, *AMER1*, and *AXIN2* were identified in SRCCs, which may also contribute to β-catenin activation in the absence of *RNF43* and *APC* mutation (Fig. S5).

MAPK and PI3K pathways are also important in the development of AC (Fearon and Vogelstein, 1990; TCGA, 2012; Sanchez-Vega et al., 2018). SRCC presented a lower mutation load in MAPK (20.7%, 60.5%, and 84.7% in SRCC, AC, and MAC respectively) and PI3K pathways (6.9%, 46.1%, and 49.2% in SRCC, AC, and MAC respectively) (Figs. 1C, S4 and S6). The mutation rates of several cancer driver genes were dramatically different across different subtypes. For instance, mutations in *PIK3CA*, *PTEN*, *IRS2* were enriched in AC and MAC, whereas almost absent in SRCC; and *KRAS* mutation rate in SRCC was about 4-fold lower than that in AC or MAC (Fig. 1D). Lower mutation rates of *PIK3CA* and *KRAS* in SRCC have also been reported recently (Korphaisarn et al., 2019). The phospho-ERK and phospho-S6 signals, indicators of MAPK and PI3K activities respectively, were much lower in SRCC, which was consistent with mutation data (Fig. 1F).

To identify subtype-specific gene expression signatures, we performed RNA-seq with SRCC, MAC and AC samples (*n* = 6 per subtype) (Table S13). The gene expression pattern of SRCC was drastically different from that of AC and MAC (Fig. 2A and Table S14), largely consistent with a previous study (Nam et al., 2018). Two mucin genes, *MUC2* and *MUC5AC*, were highly expressed in SRCC and MAC, which may contribute to elevated production of mucin (Fig. 2B). Pathway enrichment analysis showed that upregulated genes in SRCC were associated with cell adhesion and calcium signaling pathways, while downregulated genes were associated with cell cycle and cellular metabolism pathways (Fig. 2C). Similar results were obtained when SRCC and MAC expression data were compared (Fig. S7).

**Figure 2:**
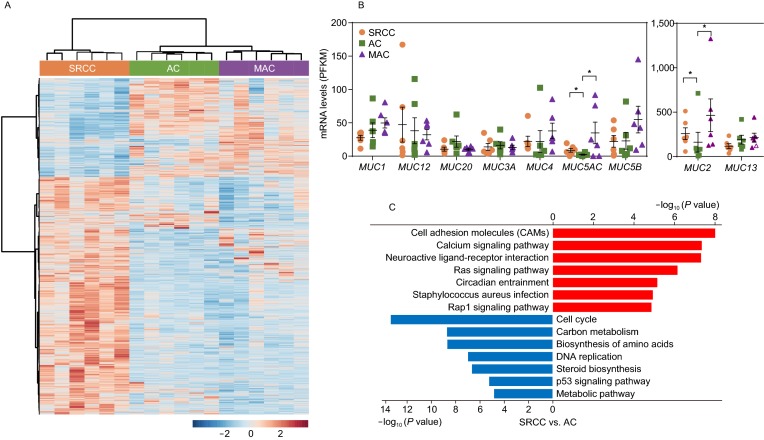

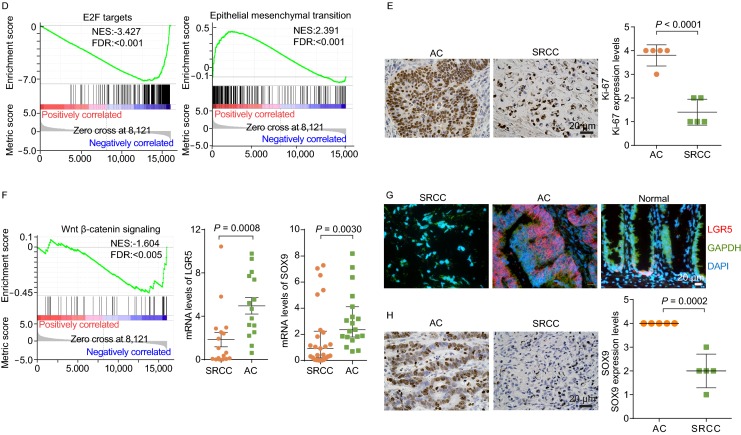
**Gene expression analysis of colorectal SRCC**. (A) Heatmap showing differentially expressed genes in SRCC, AC, and MAC (*n* = 6 per subtype, samples from FUSCC) by RNA-seq. Blue and red colors represent down- and up-regulated genes respectively. (B) Expression levels of mucin genes were compared, and *MUC2* and *MUC5AC*, were highly expressed in SRCC and MAC. RPKM data from RNA-seq was shown, * indicate *P* > 0.05 (Student’s *t* test). For *MUC2* gene, the highest data point of each group was considered as outlier and not included in analysis. (C) Kyoto encyclopedia of genes and genomes (KEGG) pathways enriched in differentially expressed genes between SRCC and AC. (D) Gene sets of E2F targets and epithelial-mesenchymal transition (EMT) were enriched in differentially expressed genes between SRCC and AC. (E) The expression (IHC, left) of proliferation marker Ki-67 in SRCC and AC, with quantification (right). (F) Targets of Wnt-β-catenin were downregulated in SRCC, and the mRNA expression of *LGR5* and *SOX9* was assessed in a larger cohort of SRCC and AC specimens by qPCR. (G) The mRNA expression of *LGR5* in SRCC, AC, and normal colon tissue assessed by ISH. (H) The protein expression of SOX9 in SRCC and AC assessed by IHC. Scale bar, 20 μm

Using the gene set enrichment analysis (GSEA), the differentially expressed genes in SRCC (compared to AC) were negatively correlated with E2F signaling, MTOR signaling, and metabolism (Figs. 2D and S8), which may underpin the slower proliferation of SRCC, as indicated by significantly lower Ki-67 expression in SRCC (Fig. 2E). On the other hand, the expression profile of SRCC was positively associated with terms of EMT and angiogenesis, which may contribute to the aggressiveness and local metastasis of SRCC. The negative association between proliferation and metastasis appears counterintuitive, however as reported previously, slow proliferation may gain ability to spread or survive outside of original microenvironment (Anjomshoaa et al., 2009).

Another term negatively associated with genes expression in SRCC was Wnt-β-catenin signaling (Fig. 2F). Many β-catenin target genes, such as *LGR5*, *SOX9*, *AXIN2*, and *MSI1*, were expressed at lower levels in SRCC compared to those in AC (Table S14). The relatively lower expression of *LGR5* and *SOX9* in SRCC was further verified by quantitative PCR, *in situ* hybridization, or IHC (Fig. 2F–H). Comparing to *APC* mutations in AC or MAC, *RNF43* mutations may moderately activate β-catenin, which in turn promote tumorigenesis of SRCC.

In summary, as indicated by genomic and transcriptomic profiling, SRCC represents a molecularly distinct subtype of CRC. Our findings will serve as a blueprint for dissecting pathogenic mechanisms of SRCC, and provide insights in developing molecularly-targeted therapies for SRCC patients.

## FOOTNOTES

This study is supported by grants from the National Natural Science Foundation of China (81622038, 31571479, and 81772965 to F.X. Y., 31470826 and 31670858 to G. H.), the National key R&D program of China (2018YFA0800304) to F.X. Y., Science and Technology Commission of Shanghai Municipality (19JC1411100 to F.X. Y., 16411966300 to G. H., 16411966300 and 18401933402 to J. P.), Shanghai Municipal Commission of Health and Family Planning (2017BR018 to F.X. Y.) and Shanghai Sailing Program (19YF1409500 to Y. L.). We would like to thank Dr. Kang Chen for proofreading of this manuscript.

G. H., J. P., and F.X. Y. designed the experiments. Specimens and clinical data collection: Y. Li, R. W., S. C., and J. P. prepared specimens and collected clinical data. H. W., and F.X. Y. performed WES. D. Z., D. D., and Y. Liu processed WES data. S. H., J. P., and F.X. Y. performed RNAseq and analysis. Y. Li, J. L., X. W., Y. W., G. F., C. F., X. M., M. P., Q. G., X. X., X. H., Y. Z., and S. M. performed experiments. Y. Li, J. L., J. G., L. Z., Q. H., and F.X. Y. analyzed data. Y. Li, J. L., L. Z., Q. H., and F.X. Y. wrote the manuscript.

All authors declare that they have no conflict of interest. This article does not contain any studies with human or animal subjects performed by the any of the authors.

## Electronic supplementary material

Below is the link to the electronic supplementary material.

Supplementary material 1 (PDF 3195 kb)

Supplementary material 2 (XLSX 8494 kb)
