# Supplementary material for: Frequent RNF43 mutation contributes to moderate activation of Wnt signaling in colorectal signet-ring cell carcinoma
Source: Protein Cell. 2020 Feb 1;11(4):292–8. doi: 10.1007/s13238-020-00691-0 (PMC7093338; doi:10.1007/s13238-020-00691-0)
Supplement: Supplementary file 1 — Supplementary material 1 (PDF 3195 kb) [file 13238_2020_691_MOESM1_ESM.pdf]

SUPPLEMENTARY METHODS

Study approval

Ethical approval was obtained from the Ethical Committee and Institutional Review Board of the Fudan University Shanghai Cancer Center (FUSCC), and informed consent was obtained from patients or legal guardians.

Cohort and survival analysis

Patients (4,044) with primary CRC underwent surgeries in FUSCC from Dec 1, 2009 to Dec 31, 2013 were enrolled in this study, including 3,762 patients with adenocarcinoma (AC), 250 patients with mucinous adenocarcinoma (MAC) and 32 patients with signet-ring cell carcinoma (SRCC) (**Table S1**). The inclusion criteria were as follows: (i) Patients were aged 18 years or older; (ii) colorectal cancer was confirmed by histopathology, including AC, MAC and SRCC without mixture of other histology; (iii) patients had not received neoadjuvant therapy; (iv) patients underwent radical resection; (v) patients were followed-up regularly with intact survival and clinicopathological information. The clinicopathological information was retrospectively collected from the prospective database of colorectal cancer. Ethical approval was obtained from the Ethical Committee and Institutional Review Board of the FUSCC, and informed consent was obtained from patients or legal guardians.

Follow-up for the patients was completed on December 31, 2014. The median length of follow-up was 27.0 months. Patients were followed up regularly according to NCCN guidelines. As this study described the prognosis of patients with CRC, analysis of OS and DFS were ascertained. The OS was defined as the time from treatment to death from any cause, and the DFS was defined as the time from treatment to the first recurrence or death. The survival data was provided by Clinical Statistics Center of FUSCC, relying on the hospital medical records follow-up platform or contacts with patients by phone or email. Patients who were alive at last follow-up were censored for analysis.

Tumor specimens

We sought to characterize the repertoire of somatic genetic alternations and expression signature of colorectal SRCCs by whole-exome sequencing (WES) and RNA-sequencing (RNA-seq), to determine their differences from colorectal adenocarcinomas (ACs) and mucinous adenocarcinomas (MACs). In total, 29 paired SRCCs and normal samples were subjected to WES and 6 samples of per subtype were subjected to RNA-seq. All CRC samples were obtained from the surgical specimens of Chinese patients treated in FUSCC from April 2008 to March 2016 (**Tables S2 and S3**). Tumor specimens fixed by formalin were sectioned, stained with hematoxylin and eosin, and inspected independently by two certified pathologists (Sheng WQ and Huang D) to determine histological subtype and tumor grade. SRCC specimens containing at least 70% signet-ring cells were included, both colorectal

tumor tissue and non-cancerous colorectal mucosa tissue microdissected within 30 min of surgical resection were stored at -80 ℃ and used for genomic DNA extraction and RNA extraction.

DNA isolation and whole-exome sequencing (WES)

Genomic DNA was extracted with QIAamp DNA Mini kits (Qiagen) from frozen tissue samples and quality controlled by using Bioanalyzer (Agilent). DNA fragments containing exome sequences were captured using the SureSelect XT Human All Exon V4 Plus library (Agilent). Constructed libraries were prepared for sequencing using Illumina's cBot cluster generation system with TruSeq PE Cluster Kit v3 (Illumina) and finally sequenced on an Illumina HiSeq X10 (Illumina) using the 150 bp paired-end mode.

TCGA Colorectal Cancer Data Sets

We retrieved raw data of CRC samples with both mutation annotation and copy number from TCGA (https://tcga-data.nci.nih.gov). Based on clinical information, all samples are AC or MAC. After matching clinical and sequencing data, we analyzed point mutations and CNVs for 458 AC and 59 MAC (**Tables S4 and S5**).

Identification of somatic alterations

Somatic short variants and CNVs were called followed the GATK Best Practices (the GATK4 pipeline). Briefly, sequencing reads were aligned to the hg38 human genome

reference sequence using BWA. Duplicated reads and base quality scores were recalibrated before the somatic mutations were called using MuTect2. Somatic alterations identified were shown in **Tables S6-7**. The SRCC sequencing coverage was >100-fold in average, with >20-fold coverage for 96.9% targeted exons. In total, we identified 9,752 non-silent somatic mutations, including 4,811 missense mutations, 225 nonsense mutations, 883 short insertion-deletion polymorphisms (indels, 195 insertions and 688 deletions), 131 splice site mutations, 1839 promoter mutations and 1,863 UTR mutations.

Mutation Burden

Tumor mutation burden (TMB) was defined as the number of nonsynonymous alterations (SNVs or indels) in coding region per Mb reads for each patient (**Table S8**). After ranking and lining each TMB point in each subtype, the sample had the largest curvature was set as cut-off. Tumor samples which had higher TMB than the cut-off point were considered as hypermutated [1]. Using this criterion, 89.7%, 62.7%, and 86.0% of SRCC, MAC, and AC respectively were grouped as non-hypermutated.

Mutation spectra and signature analysis

Mutational spectra were analyzed using the MutationalPatterns R package, release 3.4 [2] to determine genomic context of all somatic SNVs and generate mutation count matrix. The number of mutations in the 96-substitution matrix (C>A/G>T, C>G/G>C, C>T/G>A, T>A/A>T, T>C/A>G and T>G/A>C, with the bases immediately left and

right behind to each substitution in the target region) was counted for each sample (**Table S9**).

Immunohistochemistry (IHC) and In Situ Hybridization (ISH)

Tissue samples were washed with PBS, fixed in freshly prepared 4% neutral buffered paraformaldehyde, and embedded in paraffin blocks. Formalin-fixed tissues were cut into 4 μm sections and placed on polylysine-coated slides. Paraffin sections were baked for 1h at 56 ℃, de-paraffinized in xylene, rehydrated through graded ethanol, processed for antigen retrieval in 0.1 M citrate buffer (pH 6.0), and quenched for endogenous peroxidase activity in 0.3 % hydrogen peroxide for 10 min. Sections were then blocked for 1h with 10% goat serum in 0.1% PBST and incubated with primary antibody overnight at 4℃. Detailed information of primary antibodies used for IHC (anti-β-catenin, anti-pS6, anti-pERK, anti-MYC, anti-Ki-67 and anti-SOX9) was shown in **Table S15**. Signal amplification and detection were performed using the EnVision+System-HRP (AEC) (Dako, K4005) following manufacturer's instructions. All sections were counterstained with hematoxylin (Sigma) and mounted in a non-aqueous mounting medium. ISH was performed using the QuantiGene ViewRNA ISH Tissue 2-plex Assay kit (QVT0012) according to manufacturer's instructions, LGR5 (human VB1-17146) and GAPDH (human VB6-10337) probes were from Affymetrix. GAPDH ISH confirmed mRNA integrity of all cells. Hematoxylin and eosin staining were performed as described [3].

RNA isolation and quantitative real-time PCR

RNA was extracted using RNeasy mini kit (QIAGEN, 74104). A PrimeScript™ RT Master Mix (Perfect Real Time) kit (Takara, RR036A) was used to produce cDNA. qRT-PCR was performed in ABI 7900HT (Applied Biosystems) using SYBR *Premix Ex Taq* II (Tli RNaseH Plus) kit (Takara, RR820A). RNA expression was normalized to β-actin. Primers were listed in **Table S16**.

RNA-seq analysis

The total RNA was treated with Ribo-off rRNA Depletion Kit (Vazyme) before constructing the RNA-seq libraries. RNA-seq libraries were prepared using VAHTS Total RNA-seq (H/M/R) Library Prep Kit for Illumina (Vazyme) following the manufacturer's instructions. Briefly, ribosome depleted RNA samples were fragmented and then used for first- and second-strand cDNA synthesis with random hexamer primers. The cDNA fragments were treated with DNA End Repair Kit to repair the ends, then modified with Klenow to add an A at the 3' end of the DNA fragments, and finally ligated to adapters. Purified dsDNA was subjected to 12 cycles of PCR amplification, and the libraries were sequenced by Illumina sequencing platform on a 150 bp paired-end run. Sequencing reads from RNA-seq data were aligned using the spliced read aligner HISAT2, which was supplied with the Ensembl human genome assembly (Genome Reference Consortium GRCh38) as the reference genome. Gene expression levels were calculated by the FPKM (fragments per

kilobase of transcript per million mapped reads). Annotations of mRNA in the human genome were retrieved from the GENCODE (v25) database. The differentially expressed genes between each group were analyzed using wilcox test. The significant candidates were extracted with fold change (median) >2 or <0.5 and p value < 0.05. The significantly differentially expressed genes were tested for KEGG pathway using the Database for Annotation, Visualization and Integrated Discovery (DAVID) v6.8. The enriched pathways were filtered with p value <0.01. Gene Set Enrichment Analysis (GSEA) was used to investigate the enrichment of cancer hallmarks signature from MSigDB 5.0 and colon progenitor signatures defined recently [4].

Statistical analysis

Statistical analysis was performed by SPSS statistics software, version 22.0 (SPSS, Chicago, IL, USA). The continuous variables were tested for normal distribution before analyzing by one-way ANOVA test. The ranked variables were assessed by the Log-rank test or Mann-Whitney test. The categorical variables were taken with the Pearson’s Chi-square test (or Fisher’s exact test). Impact of clinical characteristics on survival outcomes were estimated by using Kaplan-Meier method. Associations of genetic alterations with clinical characteristics were estimated by logistic regression. A two-tailed *P* value of less than 0.05 was regarded as statistically significant.

SUPPLEMENTARY REFERENCES

1. TCGA. Comprehensive molecular characterization of human colon and rectal cancer. Nature 2012;487(7407):330-7.

2. Blokzijl F, Janssen R, van Boxtel R, *et al.* MutationalPatterns: comprehensive genome-wide analysis of mutational processes. Genome Med 2018;10(1):33.

3. Hua G, Wang C, Pan Y, *et al.* Distinct Levels of Radioresistance in Lgr5(+) Colonic Epithelial Stem Cells versus Lgr5(+) Small Intestinal Stem Cells. Cancer Res 2017;77(8):2124-2133.

4. Gao S, Yan L, Wang R, *et al.* Tracing the temporal-spatial transcriptome landscapes of the human fetal digestive tract using single-cell RNA-sequencing. Nat Cell Biol 2018;20(6):721-734.

SUPPLEMENTARY FIGURE LEGENDS

Figure S1. Typical histology and prognosis of SRCC, AC, and MAC. A) Representative H&E staining of normal colon, SRCC, AC and MAC. **B**) Overall survival curves of SRCC (n=32), MAC (n=250), or AC (n=3,762) patients at FUSCC. **C**) Disease-free survival curves of SRCC (n=30), MAC (n=229), or AC (n=3,348) patients without distant metastases at FUSCC. *P* value was obtained using log-rank test.

Figure S2. Nucleotide substitution analysis for SRCC (n=29), MAC (n=59) and AC (n=458). A) Mutational signatures in SRCC, MAC and AC. B) Distribution of six substitution patterns sorted by subtypes.

Figure S3. Sanger sequencing validation of critical mutated genes in SRCC. Sanger sequencing for *RNF43*, *BRAF*, *KRAS* or *SMAD4* mutated samples were performed.

Figure S4. Comparison of mutation frequencies in different signaling pathways' genes in SRCC (n=29), MAC (n=59) and AC (n=458). CRC-related pathways, including WNT, TGFβ, MAPK (RTK/RAS), PI3K, and p53 pathways, were analyzed. Blue and red colors indicate activating and inactivating mutations respectively.

Figure S5. Genomic alternation patterns in WNT pathway genes in SRCC (n=29), MAC (n=59) and AC (n=458). All samples (upper panel) or non-hypermutated samples (lower panel) were analyzed separately.

Figure S6. Genomic alternation patterns in TGF-β, MAPK, and PI3K pathways in SRCC (n=29), MAC (n=59) and AC (n=458). All samples (upper panel) or non-hypermutated samples (lower panel) were analyzed separately.

Figure S7. KEGG pathways enriched in differentially expressed genes between different subtypes of CRC. A) SRCC vs AC; **B**) SRCC vs MAC; **C**) AC vs SRCC.

Figure S8. GSEA analysis of SRCC-associated gene expression against different pathway hallmarks. Top, genes differentially expressed in SRCC and AC. Bottom, genes differentially expressed in SRCC and normal colon. Pathways included were involved in Cancer, Cell cycle, Metabolism, or Signaling. NES: Normalized Enrichment Score; FDR: False Discovery Rate.

Figure S1

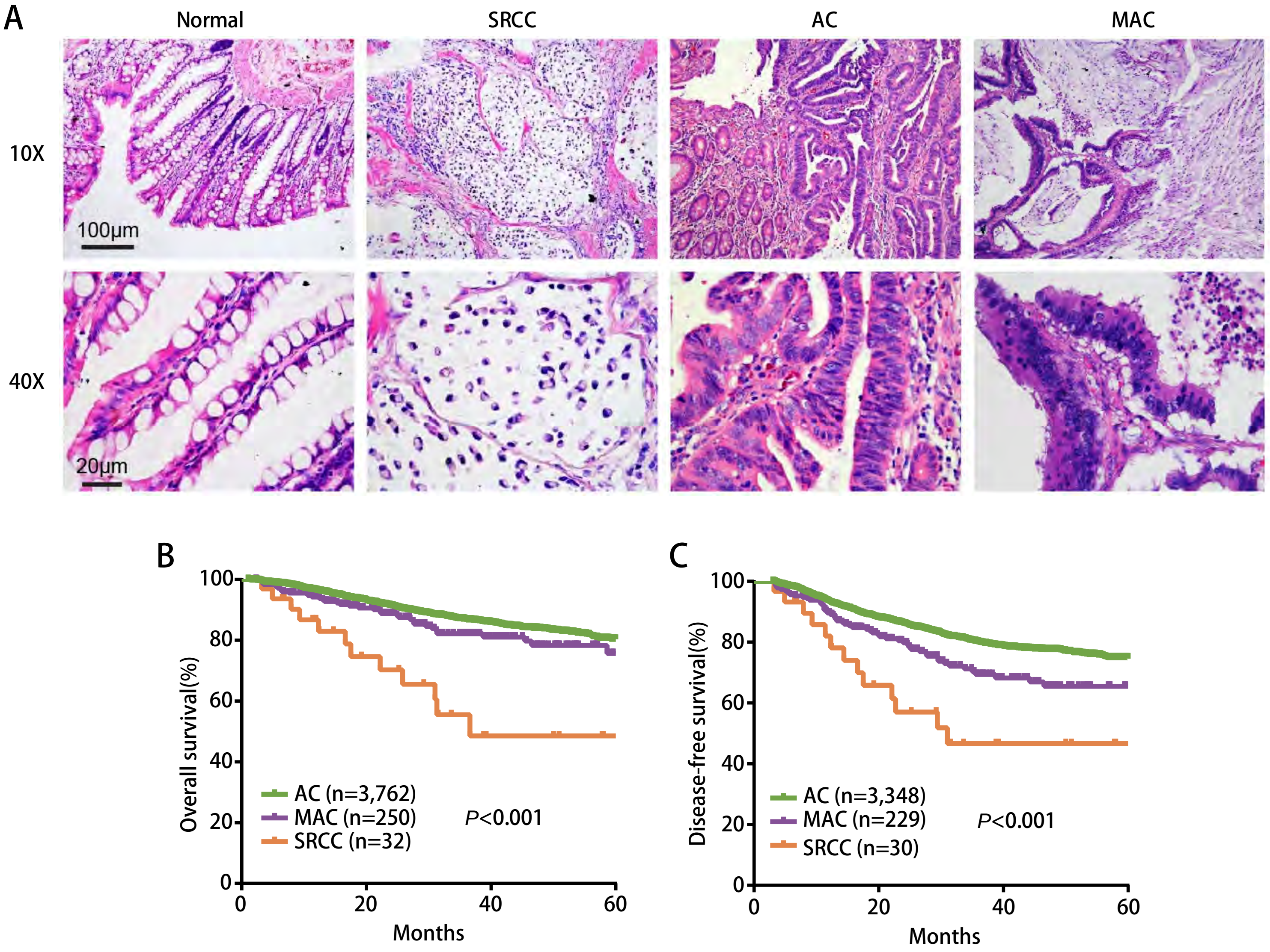
A
Normal
SRCC
AC
MAC
10X
40X
100μm
20μm
B
Overall survival(%)
Months
AC (n=3,762)
MAC (n=250)
SRCC (n=32)
P<0.001
C
Disease-free survival(%)
Months
AC (n=3,348)
MAC (n=229)
SRCC (n=30)
P<0.001

Figure S2

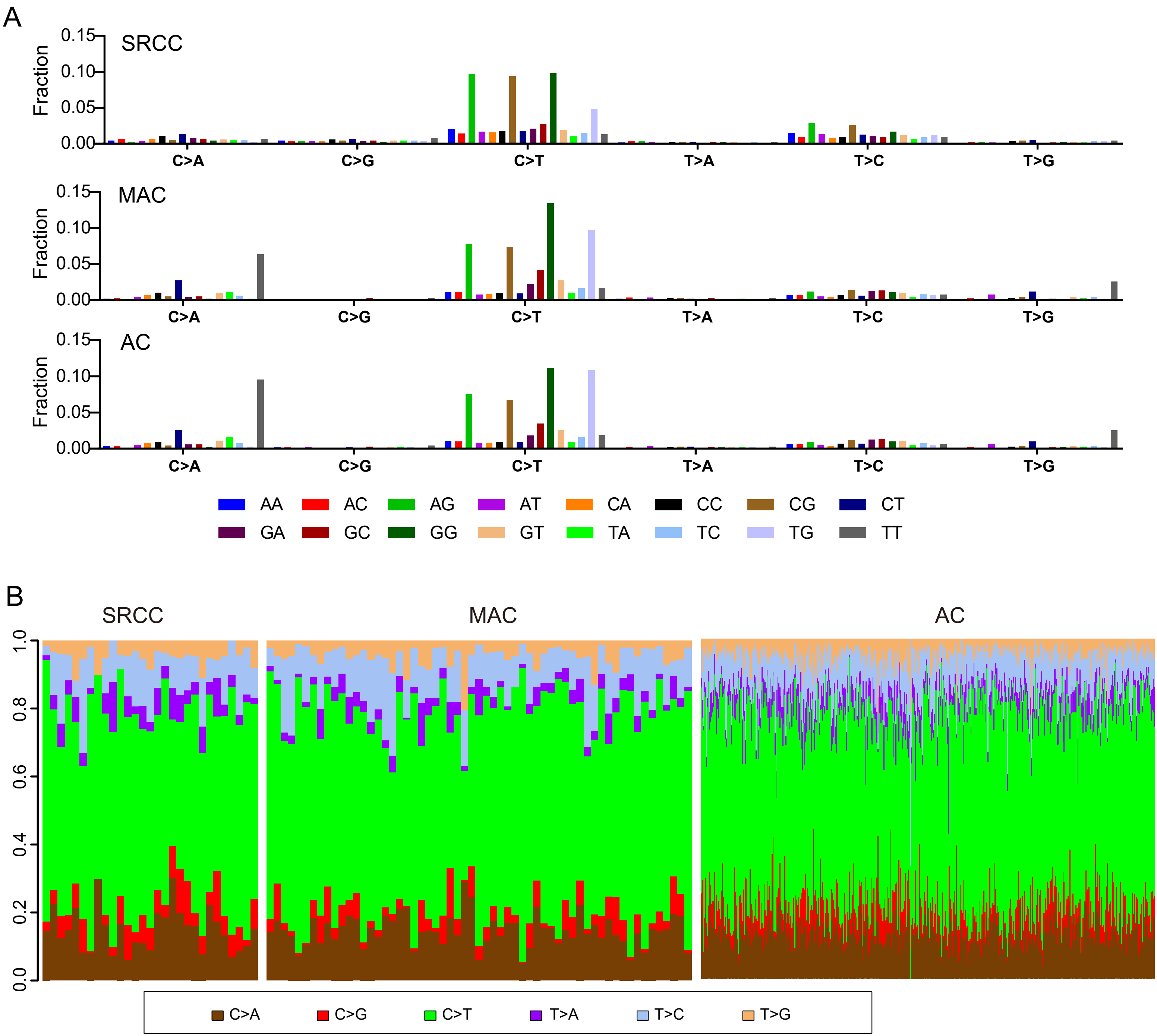
A
SRCC
MAC
AC
Fraction
0.15
0.10
0.05
0.00
C>A
C>G
C>T
T>A
T>C
T>G
AA
AC
AG
AT
CA
CC
CG
CT
GA
GC
GG
GT
TA
TC
TG
TT
B
SRCC
MAC
AC
1.0
0.8
0.6
0.4
0.2
0.0
C>A
C>G
C>T
T>A
T>C
T>G

Figure S3

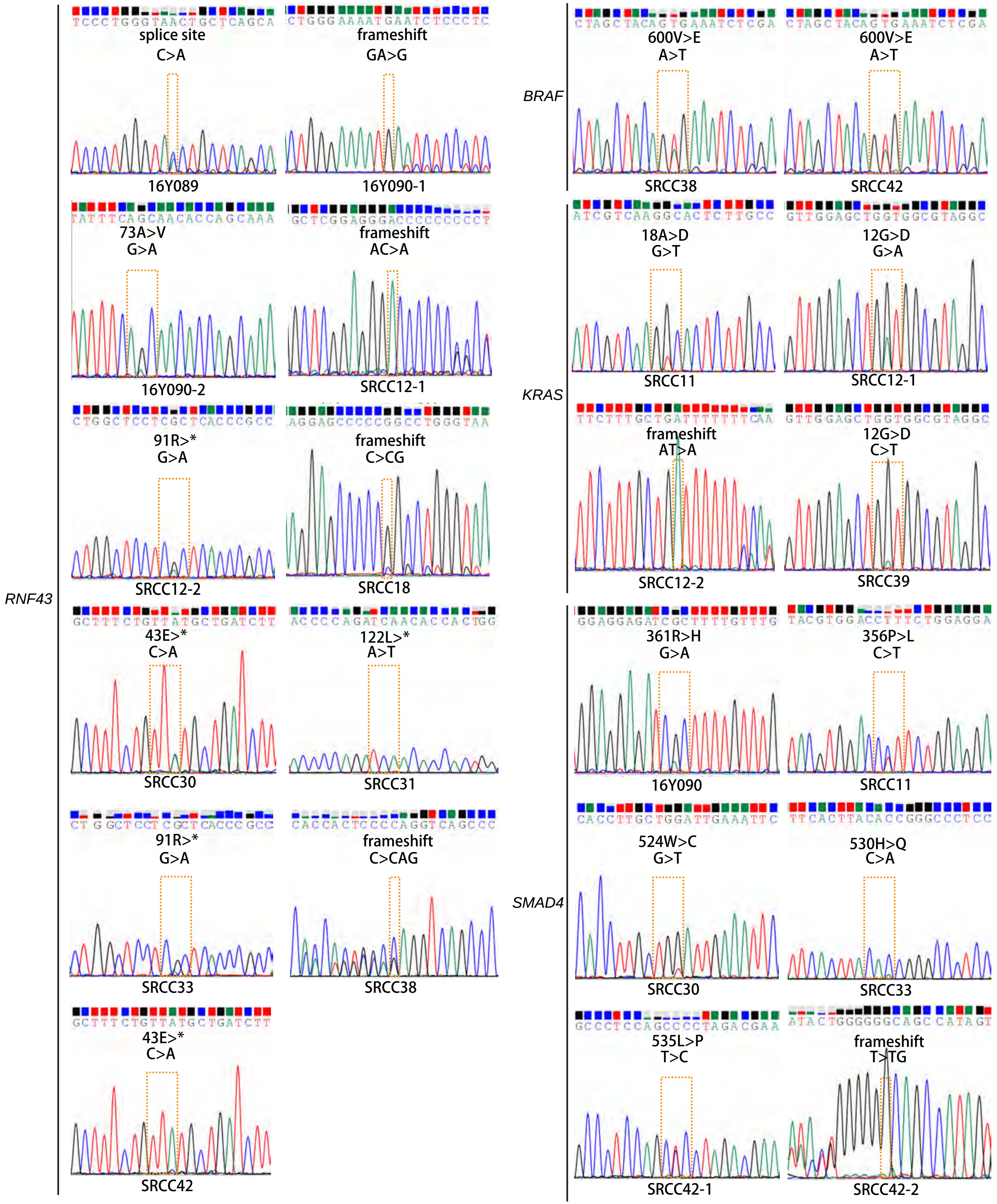

RNF43
splice site
C>A
16Y089
frameshift
GA>G
16Y090-1
73A>V
G>A
16Y090-2
frameshift
AC>A
SRCC12-1
91R>*
G>A
SRCC12-2
frameshift
C>CG
SRCC18
43E>*
C>A
SRCC30
122L>*
A>T
SRCC31
91R>*
G>A
SRCC33
frameshift
C>CAG
SRCC38
43E>*
C>A
SRCC42
BRAF
600V>E
A>T
SRCC38
600V>E
A>T
SRCC42
KRAS
18A>D
G>T
SRCC11
12G>D
G>A
SRCC12-1
frameshift
AT>A
SRCC12-2
12G>D
C>T
SRCC39
SMAD4
361R>H
G>A
16Y090
356P>L
C>T
SRCC11
524W>C
G>T
SRCC30
530H>Q
C>A
SRCC33
535L>P
T>C
SRCC42-1
frameshift
T>TG
SRCC42-2

Figure S4

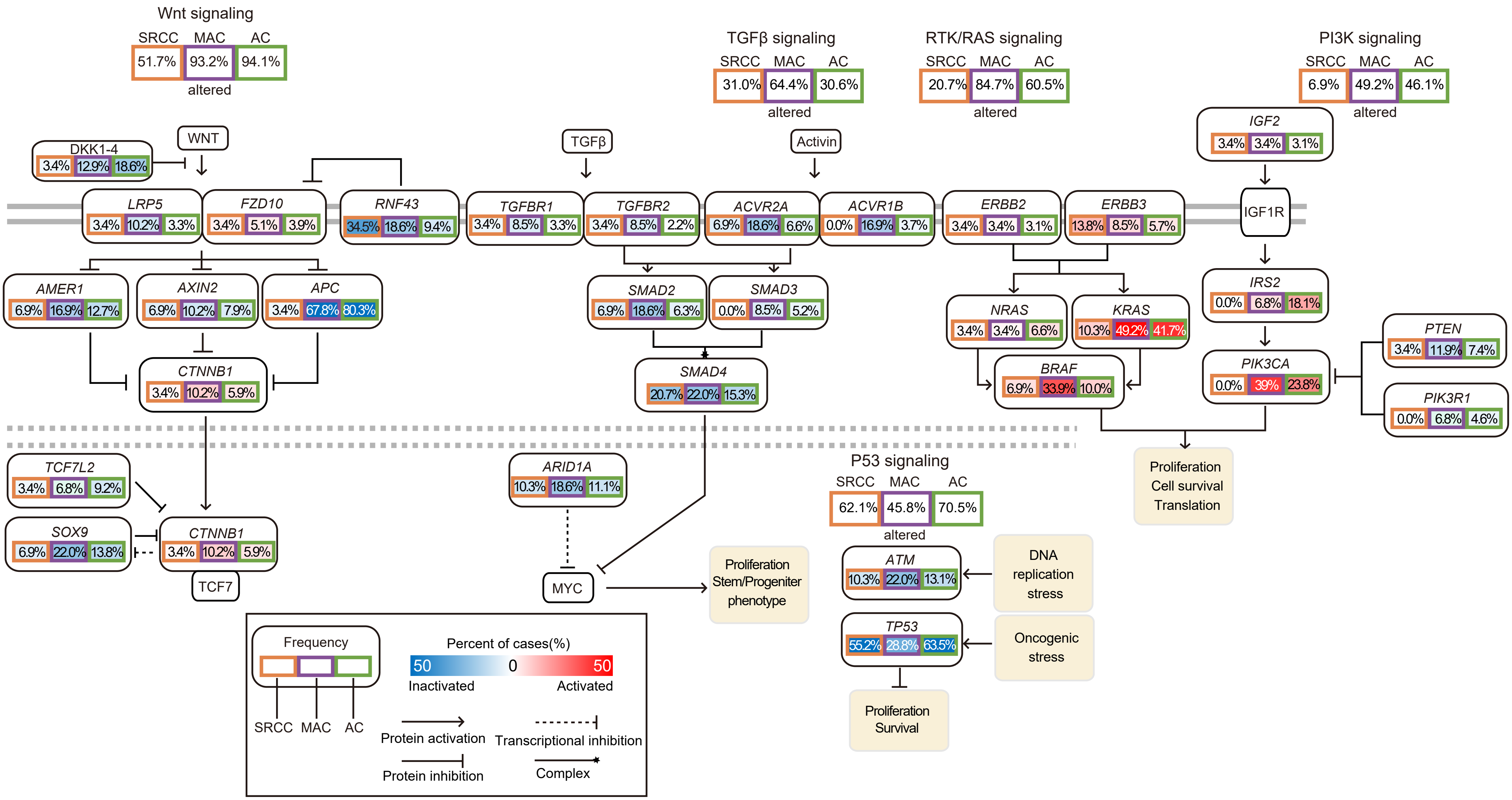
Wnt signaling
SRCC MAC AC
51.7% 93.2% 94.1%
altered
TGFβ signaling
SRCC MAC AC
31.0% 64.4% 30.6%
altered
RTK/RAS signaling
SRCC MAC AC
20.7% 84.7% 60.5%
altered
PI3K signaling
SRCC MAC AC
6.9% 49.2% 46.1%
altered
DKK1-4 3.4% 12.9% 18.6%
WNT
TGFβ
Activin
IGF2 3.4% 3.4% 3.1%
LRP5 3.4% 10.2% 3.3%
FZD10 3.4% 5.1% 3.9%
RNF43 34.5% 18.6% 9.4%
TGFBR1 3.4% 8.5% 3.3%
TGFBR2 3.4% 8.5% 2.2%
ACVR2A 6.9% 18.6% 6.6%
ACVR1B 0.0% 16.9% 3.7%
ERBB2 3.4% 3.4% 3.1%
ERBB3 13.8% 8.5% 5.7%
IGF1R
AMER1 6.9% 16.9% 12.7%
AXIN2 6.9% 10.2% 7.9%
APC 3.4% 67.8% 80.3%
SMAD2 6.9% 18.6% 6.3%
SMAD3 0.0% 8.5% 5.2%
NRAS 3.4% 3.4% 6.6%
KRAS 10.3% 49.2% 41.7%
IRS2 0.0% 6.8% 18.1%
PTEN 3.4% 11.9% 7.4%
CTNNB1 3.4% 10.2% 5.9%
SMAD4 20.7% 22.0% 15.3%
BRAF 6.9% 33.9% 10.0%
PIK3CA 0.0% 39% 23.8%
PIK3R1 0.0% 6.8% 4.6%
TCF7L2 3.4% 6.8% 9.2%
ARID1A 10.3% 18.6% 11.1%
P53 signaling
SRCC MAC AC
62.1% 45.8% 70.5%
altered
Proliferation
Cell survival
Translation
SOX9 6.9% 22.0% 13.8%
CTNNB1 3.4% 10.2% 5.9%
TCF7
MYC
Proliferation
Stem/Progeniter
phenotype
ATM 10.3% 22.0% 13.1%
DNA replication stress
TP53 55.2% 28.8% 63.5%
Oncogenic stress
Proliferation
Survival
Frequency
SRCC MAC AC
Percent of cases(%)
50 0 50
Inactivated Activated
Protein activation
Transcriptional inhibition
Protein inhibition
Complex

Figure S5

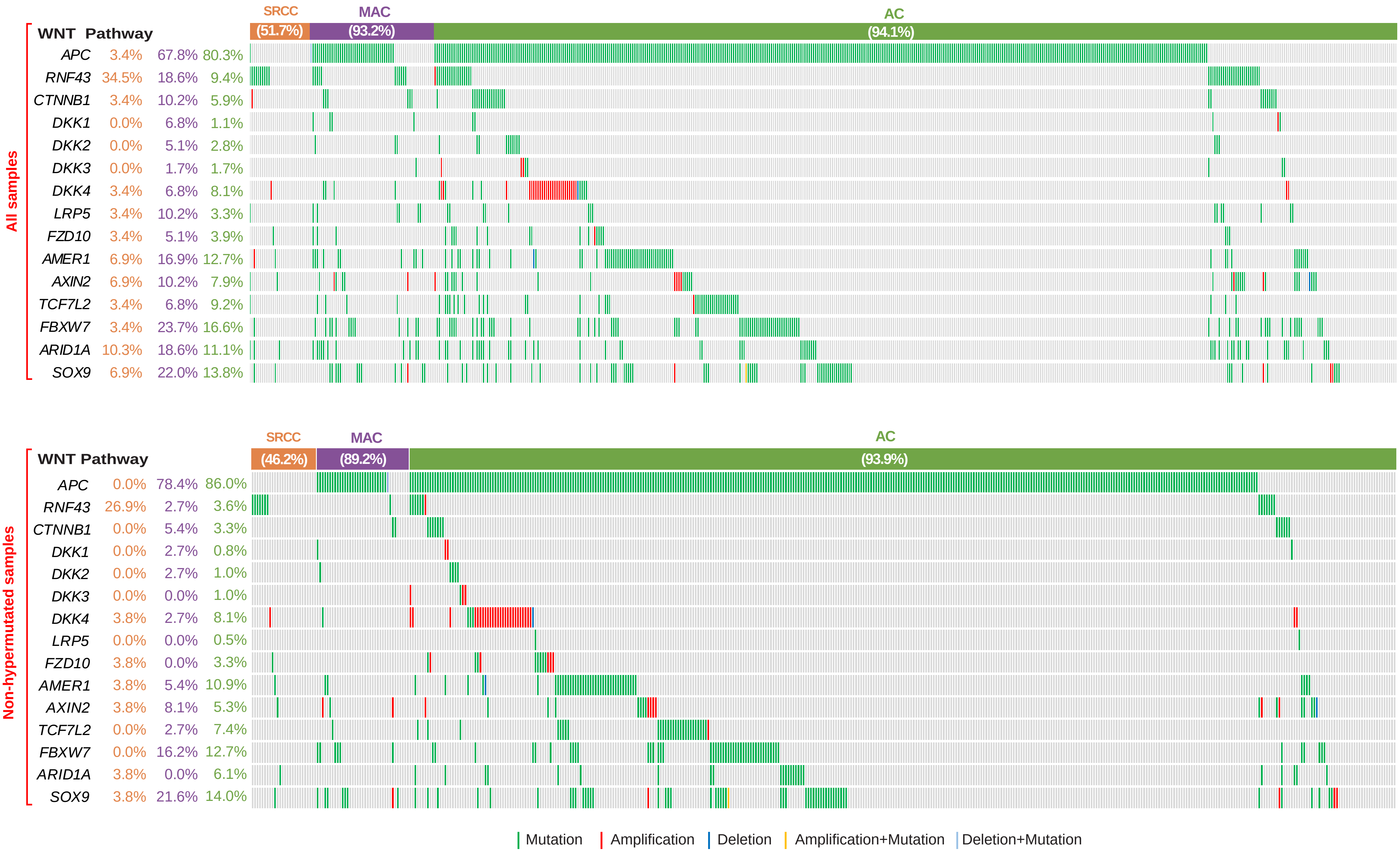

All samples
SRCC (51.7%)
MAC (93.2%)
AC (94.1%)
WNT Pathway
APC 3.4% 67.8% 80.3%
RNF43 34.5% 18.6% 9.4%
CTNNB1 3.4% 10.2% 5.9%
DKK1 0.0% 6.8% 1.1%
DKK2 0.0% 5.1% 2.8%
DKK3 0.0% 1.7% 1.7%
DKK4 3.4% 6.8% 8.1%
LRP5 3.4% 10.2% 3.3%
FZD10 3.4% 5.1% 3.9%
AMER1 6.9% 16.9% 12.7%
AXIN2 6.9% 10.2% 7.9%
TCF7L2 3.4% 6.8% 9.2%
FBXW7 3.4% 23.7% 16.6%
ARID1A 10.3% 18.6% 11.1%
SOX9 6.9% 22.0% 13.8%
Non-hypermutated samples
SRCC (46.2%)
MAC (89.2%)
AC (93.9%)
WNT Pathway
APC 0.0% 78.4% 86.0%
RNF43 26.9% 2.7% 3.6%
CTNNB1 0.0% 5.4% 3.3%
DKK1 0.0% 2.7% 0.8%
DKK2 0.0% 2.7% 1.0%
DKK3 0.0% 0.0% 1.0%
DKK4 3.8% 2.7% 8.1%
LRP5 0.0% 0.0% 0.5%
FZD10 3.8% 0.0% 3.3%
AMER1 3.8% 5.4% 10.9%
AXIN2 3.8% 8.1% 5.3%
TCF7L2 0.0% 2.7% 7.4%
FBXW7 0.0% 16.2% 12.7%
ARID1A 3.8% 0.0% 6.1%
SOX9 3.8% 21.6% 14.0%
Mutation
Amplification
Deletion
Amplification+Mutation
Deletion+Mutation

Figure S6

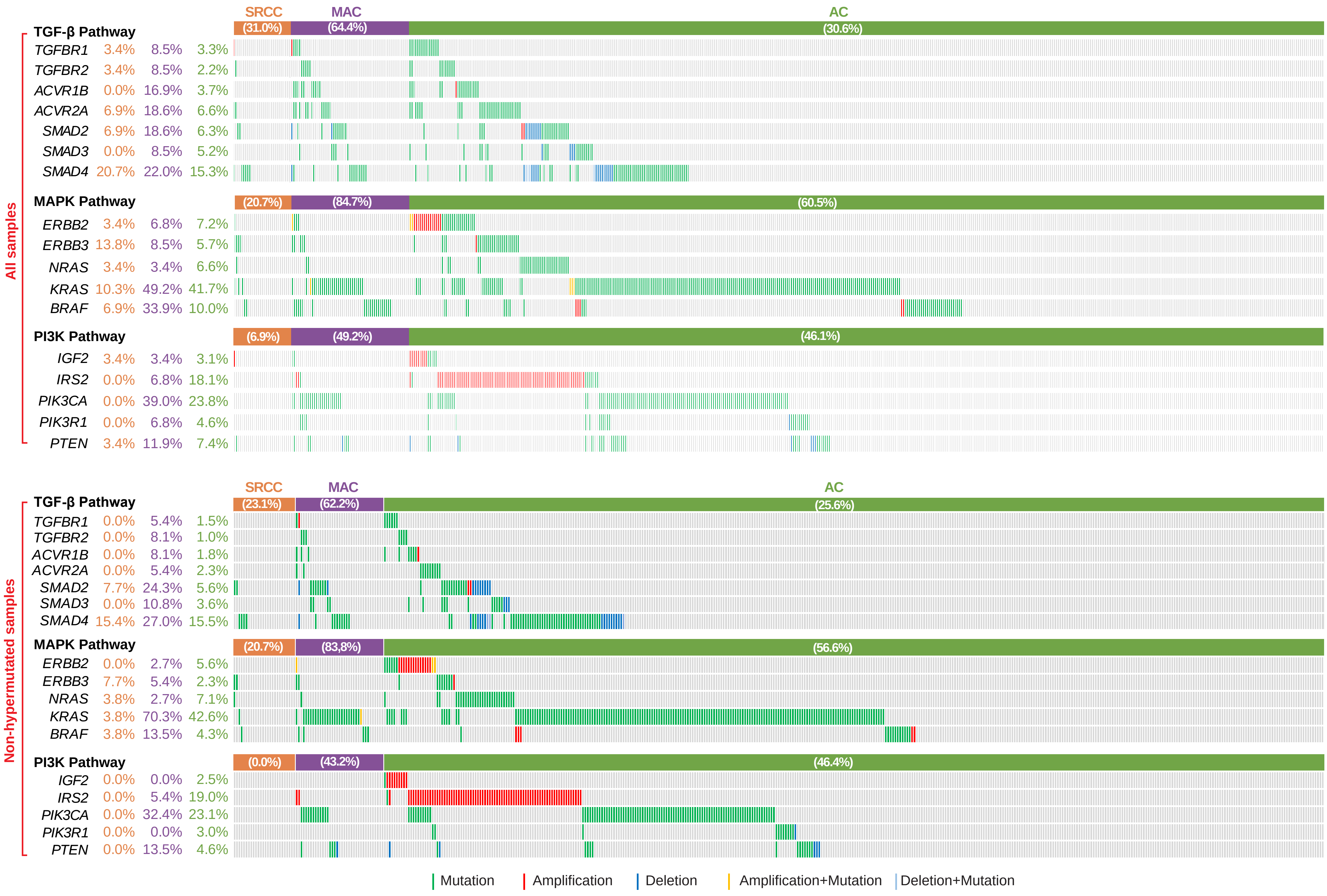

All samples
SRCC
MAC
AC
TGF-β Pathway
(31.0%)
(64.4%)
(30.6%)
TGFBR1 3.4% 8.5% 3.3%
TGFBR2 3.4% 8.5% 2.2%
ACVR1B 0.0% 16.9% 3.7%
ACVR2A 6.9% 18.6% 6.6%
SMAD2 6.9% 18.6% 6.3%
SMAD3 0.0% 8.5% 5.2%
SMAD4 20.7% 22.0% 15.3%
MAPK Pathway
(20.7%)
(84.7%)
(60.5%)
ERBB2 3.4% 6.8% 7.2%
ERBB3 13.8% 8.5% 5.7%
NRAS 3.4% 3.4% 6.6%
KRAS 10.3% 49.2% 41.7%
BRAF 6.9% 33.9% 10.0%
PI3K Pathway
(6.9%)
(49.2%)
(46.1%)
IGF2 3.4% 3.4% 3.1%
IRS2 0.0% 6.8% 18.1%
PIK3CA 0.0% 39.0% 23.8%
PIK3R1 0.0% 6.8% 4.6%
PTEN 3.4% 11.9% 7.4%
Non-hypermutated samples
SRCC
MAC
AC
TGF-β Pathway
(23.1%)
(62.2%)
(25.6%)
TGFBR1 0.0% 5.4% 1.5%
TGFBR2 0.0% 8.1% 1.0%
ACVR1B 0.0% 8.1% 1.8%
ACVR2A 0.0% 5.4% 2.3%
SMAD2 7.7% 24.3% 5.6%
SMAD3 0.0% 10.8% 3.6%
SMAD4 15.4% 27.0% 15.5%
MAPK Pathway
(20.7%)
(83,8%)
(56.6%)
ERBB2 0.0% 2.7% 5.6%
ERBB3 7.7% 5.4% 2.3%
NRAS 3.8% 2.7% 7.1%
KRAS 3.8% 70.3% 42.6%
BRAF 3.8% 13.5% 4.3%
PI3K Pathway
(0.0%)
(43.2%)
(46.4%)
IGF2 0.0% 0.0% 2.5%
IRS2 0.0% 5.4% 19.0%
PIK3CA 0.0% 32.4% 23.1%
PIK3R1 0.0% 0.0% 3.0%
PTEN 0.0% 13.5% 4.6%
Mutation
Amplification
Deletion
Amplification+Mutation
Deletion+Mutation

Figure S7

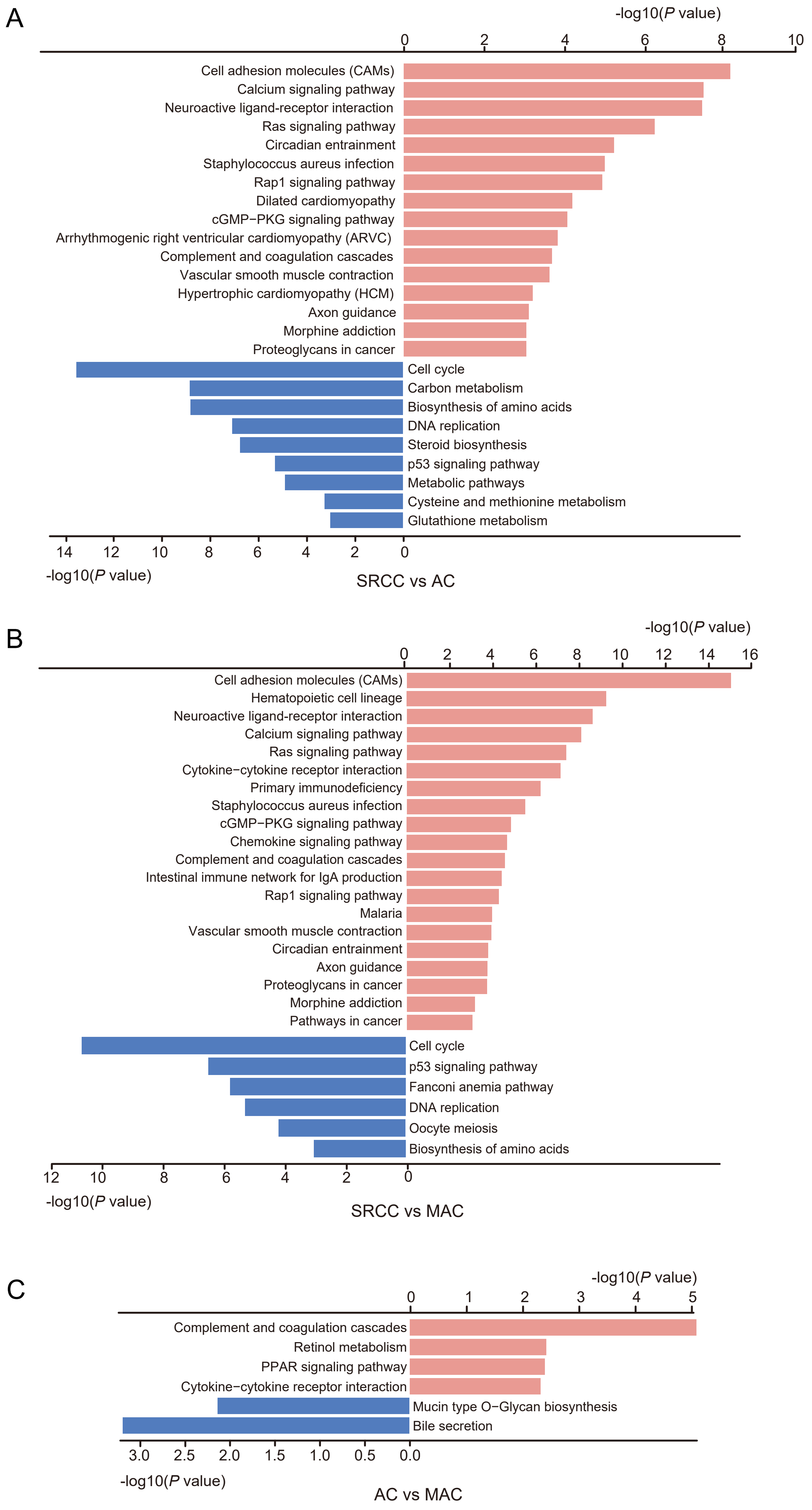

A
-log10(P value)
0 2 4 6 8 10
Cell adhesion molecules (CAMs)
Calcium signaling pathway
Neuroactive ligand-receptor interaction
Ras signaling pathway
Circadian entrainment
Staphylococcus aureus infection
Rap1 signaling pathway
Dilated cardiomyopathy
cGMP−PKG signaling pathway
Arrhythmogenic right ventricular cardiomyopathy (ARVC)
Complement and coagulation cascades
Vascular smooth muscle contraction
Hypertrophic cardiomyopathy (HCM)
Axon guidance
Morphine addiction
Proteoglycans in cancer
Cell cycle
Carbon metabolism
Biosynthesis of amino acids
DNA replication
Steroid biosynthesis
p53 signaling pathway
Metabolic pathways
Cysteine and methionine metabolism
Glutathione metabolism
14 12 10 8 6 4 2 0
-log10(P value)
SRCC vs AC
B
-log10(P value)
0 2 4 6 8 10 12 14 16
Cell adhesion molecules (CAMs)
Hematopoietic cell lineage
Neuroactive ligand-receptor interaction
Calcium signaling pathway
Ras signaling pathway
Cytokine−cytokine receptor interaction
Primary immunodeficiency
Staphylococcus aureus infection
cGMP−PKG signaling pathway
Chemokine signaling pathway
Complement and coagulation cascades
Intestinal immune network for IgA production
Rap1 signaling pathway
Malaria
Vascular smooth muscle contraction
Circadian entrainment
Axon guidance
Proteoglycans in cancer
Morphine addiction
Pathways in cancer
Cell cycle
p53 signaling pathway
Fanconi anemia pathway
DNA replication
Oocyte meiosis
Biosynthesis of amino acids
12 10 8 6 4 2 0
-log10(P value)
SRCC vs MAC
C
-log10(P value)
0 1 2 3 4 5
Complement and coagulation cascades
Retinol metabolism
PPAR signaling pathway
Cytokine−cytokine receptor interaction
Mucin type O−Glycan biosynthesis
Bile secretion
3.0 2.5 2.0 1.5 1.0 0.5 0.0
-log10(P value)
AC vs MAC

Figure S8

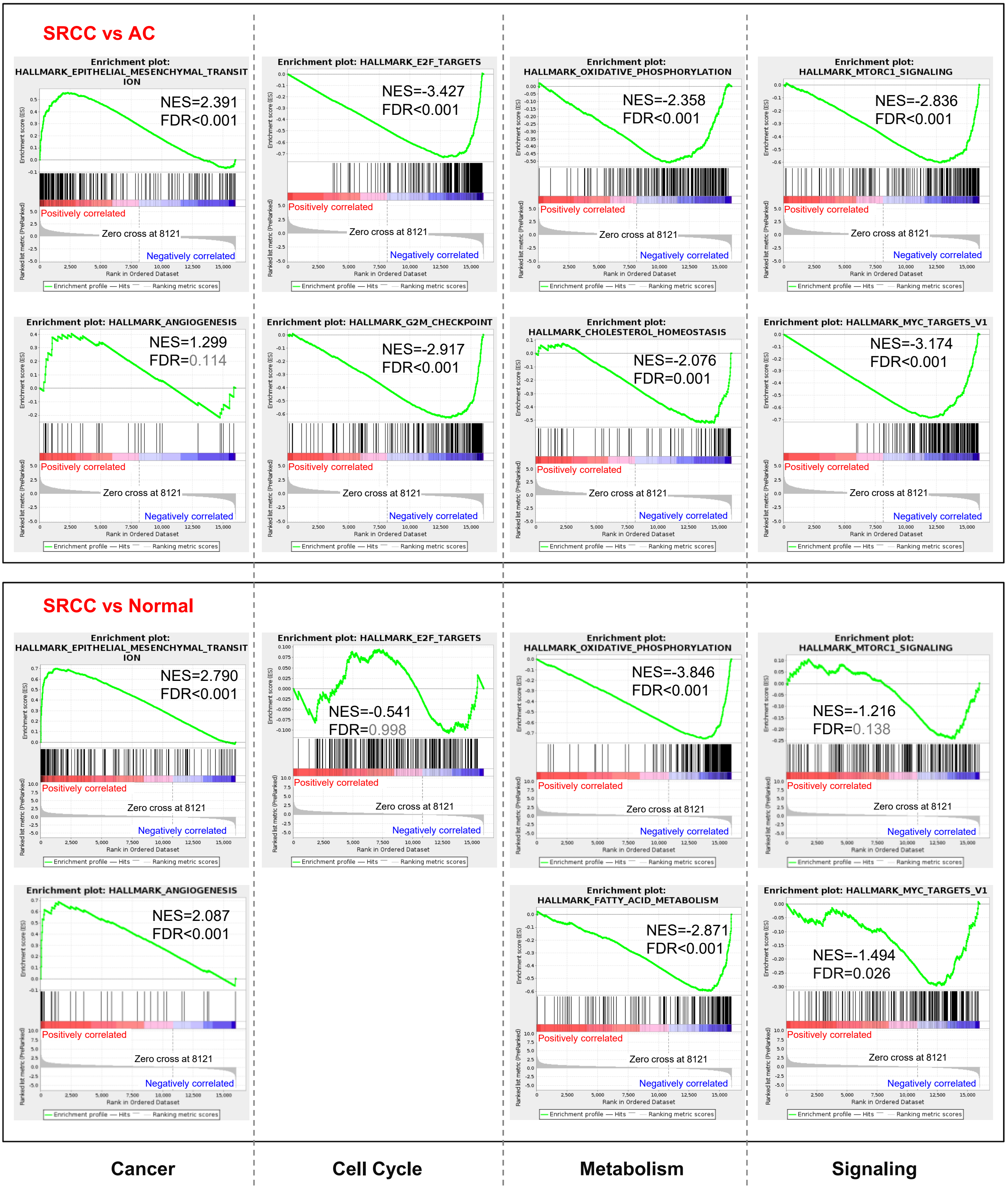
SRCC vs AC
Enrichment plot: HALLMARK_EPITHELIAL_MESENCHYMAL_TRANSITION
NES=2.391
FDR<0.001
Enrichment plot: HALLMARK_E2F_TARGETS
NES=-3.427
FDR<0.001
Enrichment plot: HALLMARK_OXIDATIVE_PHOSPHORYLATION
NES=-2.358
FDR<0.001
Enrichment plot: HALLMARK_MTORC1_SIGNALING
NES=-2.836
FDR<0.001
Enrichment plot: HALLMARK_ANGIOGENESIS
NES=1.299
FDR=0.114
Enrichment plot: HALLMARK_G2M_CHECKPOINT
NES=-2.917
FDR<0.001
Enrichment plot: HALLMARK_CHOLESTEROL_HOMEOSTASIS
NES=-2.076
FDR=0.001
Enrichment plot: HALLMARK_MYC_TARGETS_V1
NES=-3.174
FDR<0.001
SRCC vs Normal
Enrichment plot: HALLMARK_EPITHELIAL_MESENCHYMAL_TRANSITION
NES=2.790
FDR<0.001
Enrichment plot: HALLMARK_E2F_TARGETS
NES=-0.541
FDR=0.998
Enrichment plot: HALLMARK_OXIDATIVE_PHOSPHORYLATION
NES=-3.846
FDR<0.001
Enrichment plot: HALLMARK_MTORC1_SIGNALING
NES=-1.216
FDR=0.138
Enrichment plot: HALLMARK_ANGIOGENESIS
NES=2.087
FDR<0.001
Enrichment plot: HALLMARK_FATTY_ACID_METABOLISM
NES=-2.871
FDR<0.001
Enrichment plot: HALLMARK_MYC_TARGETS_V1
NES=-1.494
FDR=0.026
Positively correlated
Zero cross at 8121
Negatively correlated
Rank in Ordered Dataset
Enrichment profile — Hits — Ranking metric scores
Cancer
Cell Cycle
Metabolism
Signaling
